# Identification of ChIP-seq and RIME grade antibodies for Estrogen Receptor alpha

**DOI:** 10.1371/journal.pone.0215340

**Published:** 2019-04-10

**Authors:** Silvia-E. Glont, Evangelia K. Papachristou, Ashley Sawle, Kelly A. Holmes, Jason S. Carroll, Rasmus Siersbaek

**Affiliations:** Cancer Research UK Cambridge Institute, University of Cambridge, Robinson Way, Cambridge, United Kingdom; Universita degli Studi di Salerno, ITALY

## Abstract

Estrogen Receptor alpha (ERα) plays a major role in most breast cancers, and it is the target of endocrine therapies used in the clinic as standard of care for women with breast cancer expressing this receptor. The two methods ChIP-seq (chromatin immunoprecipitation coupled with deep sequencing) and RIME (Rapid Immunoprecipitation of Endogenous Proteins) have greatly improved our understanding of ERα function during breast cancer progression and in response to anti-estrogens. A critical component of both ChIP-seq and RIME protocols is the antibody that is used against the bait protein. To date, most of the ChIP-seq and RIME experiments for the study of ERα have been performed using the sc-543 antibody from Santa Cruz Biotechnology. However, this antibody has been discontinued, thereby severely impacting the study of ERα in normal physiology as well as diseases such as breast cancer and ovarian cancer. Here, we compare the sc-543 antibody with other commercially available antibodies, and we show that 06–935 (EMD Millipore) and ab3575 (Abcam) antibodies can successfully replace the sc-543 antibody for ChIP-seq and RIME experiments.

## Introduction

In the last decades, there has been significant interest in studying Estrogen Receptor alpha (ERα) due to its causal role in more than three quarters of breast cancers[1]. Its key role in breast cancer progression makes ERα the major target for endocrine therapies, which have substantially improved patient survival. However, resistance to these therapies occurs in many patients[2], which leads to incurable metastatic disease. Therefore, it is important to understand the mechanisms underlying ERα action in cancer initiation as well as progression of the disease. In addition, ERα plays an important role in development[3] and other diseases such as ovarian cancer[4].

Our understanding of ERα-mediated gene transcription has evolved in recent years, due to delineation of ERα-chromatin binding mechanisms through ChIP-seq (chromatin immunoprecipitation followed by next generation sequencing) experiments[5–15]. It is now clear that differential binding of ERα to chromatin is associated with clinical outcome in primary ERα-positive breast tumours[5], suggesting that changes in ERα binding mediates the altered gene expression program that dictates endocrine responsiveness and clinical outcome. In addition to changes in binding to chromatin, ERα transcriptional activity can be modulated by its association with different co-regulators and other associated transcription factors. Our lab has previously developed a method termed RIME (Rapid Immunoprecipitation of Endogenous Proteins) for the study of protein complexes using mass spectrometry[16, 17]. A key component of ERα ChIP-seq and RIME assays is the antibody that specifically and with high sensitivity targets ERα. Most ChIP-seq and RIME experiments have been performed using the ERα antibody sc-543 from Santa Cruz Biotechnology[5, 9, 17–21]. This antibody has recently been discontinued, impacting the ability to study ERα function in breast cancer as well as in other diseases and physiological conditions. Here, we compare the sc-543 (Santa Cruz Biotechnology) with other commercially available antibodies using breast cancer cells as a model and demonstrate that 06–935 (EMD Millipore) and ab3575 (Abcam) antibodies can replace sc-543 in ChIP-seq and RIME assays.

## Materials and methods

### Cell culture

MCF7 cells were cultured in Dulbecco’s Modified Eagle Medium DMEM (Gibco, Thermo Scientific) and MDA-MB-231 cells were grown in RPMI-1640 medium (Gibco, Thermo Scientific). Both media conditions were supplemented with 10% foetal bovine serum (FBS), 50 U/ml penicillin, 50 μg/ml streptomycin and 2 mM L-glutamine. Cell lines were obtained from ATCC (Middlesex). For both ChIP-seq and RIME experiments, 2x10^6^ cells were seeded in 15 cm^2^ plates and collected at 80–90% confluency.

### ChIP-Seq and RIME assays

The sc-543 (Santa Cruz), ab80922 (Abcam), ab3575 (Abcam), sc-514857 (C-3) (Santa Cruz Biotechnology), C15100066 (Diagenode) and 06–935 (EMD Millipore) antibodies were used for ChIP-qPCR. The sc-543, ab3575 and 06–935 antibodies were then used for ChIP-seq and RIME. For each ChIP, 10μg of each of the antibodies sc-543, 06–935 and ab3575 or the rabbit IgG ab37415 (Abcam) were used together with 100μl of Dynabeads Protein A (Invitrogen). The antibody and the beads were incubated overnight at 4°C with rotation. MCF7 cells were fixed for 10 minutes using 1% formaldehyde (Thermo, #28908) and quenched with 0.1M glycine. Cells were then washed and harvested in ice-cold PBS containing protease inhibitors (Roche). In order to enrich for the nuclear fraction, pellets were resuspended in Lysis Buffer 1 (50mM Hepes–KOH, pH 7.5, 140mM NaCl, 1mM EDTA, 10% Glycerol, 0.5% NP-40/Igepal CA-630, 0.25% Triton X-100) and rotated for 10 minutes, at 4°C. Cells were then pelleted, resuspended in Lysis buffer 2 (10mM Tris–HCL, pH8.0, 200mM NaCl, 1mM EDTA, 0.5mM EGTA) and incubated for 5 minutes, at 4°C with rotation. For both ChIP-seq and RIME experiments, cells were pelleted, resuspended in 300 μl Lysis buffer 3 (10mM Tris–HCl, pH 8, 100mM NaCl, 1mM EDTA, 0.5mM EGTA, 0.1% Na–Deoxycholate) and sonicated using the Bioruptor Pico sonicator (Diagenode, Liege, Belgium) for 10 cycles (30 seconds on, 30 seconds off). After sonication the samples were centrifuged at maximum speed for 10 minutes at 4°C and a small aliquot of supernatant was kept as input for ChIP-seq. The rest of the supernatant was added to the Protein A Dynabeads, which were incubated overnight with antibody. The next day, the beads for ChIP-seq were washed six times with RIPA buffer (150mM NaCl, 10mM Tris, pH 7.2, 0.1% SDS, 1% Triton X-100, 1% NaDeoxycholate), followed by one wash with TE (pH 7.4). Both ChIP samples and inputs were then de-crosslinked by adding 200 μl elution buffer (1% SDS, 0.1 M NaHCO_3_) overnight at 65°C. After reverse crosslinking, DNA was purified using the phenol-chloroform-isoamyl DNA extraction method. ChIP-seq and the input libraries were prepared using the ThruPlex Sample Prep Kit (Illumina). ERα ChIP-seq was performed in at least duplicates for each condition. For RIME, the antibody-bound beads incubated with the chromatin samples were washed 10 times with RIPA buffer and twice with 100mM AMBIC (ammonium bicarbonate) prior to mass spectrometry analysis.

### Sample preparation, LC-MS/MS analysis and data processing

A 10μL trypsin solution (15ng/ul) (Pierce) prepared in 100mM AMBIC was added to the beads followed by overnight incubation at 37°C. The next day, trypsin solution was added for a second digestion step followed by incubation for 4h at 37°C. At the end of the second step digestion, the tubes were placed on a magnet and the supernatant solution was collected and acidified by the addition of 2μl 5% formic acid. The peptides were cleaned with the Ultra-Micro C18 Spin Columns (Harvard Apparatus) and were analysed in the Dionex Ultimate 3000 UHPLC system coupled with the Q-Exactive HF (Thermo Scientific) mass spectrometer. Samples were loaded on the Acclaim PepMap 100, 100μm × 2cm C18, 5μm, 100Ȧ trapping column with the ulPickUp injection method at loading flow rate 5μL/min for 10 min. For the peptide separation the EASY-Spray analytical column 75μm × 25cm, C18, 2μm, 100 Ȧ was used for multi-step gradient elution. Mobile phase (A) was composed of 2% acetonitrile, 0.1% formic acid, 5% dimethyl sulfoxide (DMSO) and mobile phase (B) was composed of 80% acetonitrile, 0.1% formic acid, 5% DMSO. The full scan was performed in the Orbitrap in the range of 400-1600m/z at 60K resolution. For MS2, the 10 most intense fragments were selected at resolution 30K. A 2.0Th isolation window was used and the HCD collision energy was set up at 28%. The HCD tandem mass spectra were processed with the SequestHT search engine on Proteome Discoverer 2.2 software. The node for SequestHT included the following parameters: Precursor Mass Tolerance 20ppm, Maximum Missed Cleavages sites 2, Fragment Mass Tolerance 0.02Da and Dynamic Modifications were Oxidation of M (+15.995Da) and Deamidation of N, Q (+0.984Da). The Minora Feature Detector node was used for label-free quantification and the consensus workflow included the Feature Mapper and the Precursor Ion Quantifier nodes using intensity for the precursor quantification. The protein intensities were normalized by the summed intensity separately for the IgG and ERα pull downs (within group normalization). The plots for ERα coverage were created using the qPLEXanalyzer tool[22]. Heatmaps and PCA plot were done with the Phantasus Web tool (https://artyomovlab.wustl.edu/phantasus/). The mass spectrometry proteomics data have been deposited to the ProteomeXchange Consortium via the PRIDE[23] partner repository with the dataset identifier PXD012930.

### ChIP-seq data analysis

Reads were mapped to the GRCh38 genome using bwa version 0.7.12[24]. Prior to peak calling, reads were filtered according to four criteria: (1) only reads aligning to canonical chromosomes (1–22, X, Y, MT) were considered for further analysis; (2) read aligning in blacklisted regions were excluded[25]; (3) grey lists were generated using the R package GreyListChIP and reads aligned in these regions were excluded; (4) reads with a mapping quality of less than 15 were excluded. Peak calling was carried out on each ChIP sample with MACS2 version 2.1.1.20160309 using the relevant input sample[26]. Peaks with a q-value < 0.01 were accepted for further analysis. To create tag heatmaps, a consensus peak set was generated using the R package DiffBind[5, 16]. The consensus peak set was composed of any peak that was called in at least two samples. Motif analysis was carried out using AME[27] from the MEME suite version 4.12.0[28] and the HOCOMOCO Human (v10) motif database[29]. Sequences for motif analysis for each sample were derived by selecting the top 1000 peaks by q-value from the MACS2 peak set and then extracting the genomic sequence 500 bases either side of the peak summits. A detailed description of the pipeline can be found in **S1 File.** ChIP-seq data have been deposited in NCBI's Gene Expression Omnibus[30] and are accessible through GEO Series accession number GSE128208.

## Results and discussion

### ChIP-sequencing validates 06–935 and ab3575 as specific ERα antibodies

Given the discontinuation of anti-ERα antibody sc-543, we sought to validate alternatives for immunoprecipitation experiments. We first compared the established sc-543 (Santa Cruz Biotechnology) antibody with ab80922 (Abcam), ab3575 (Abcam), sc-514857 (C-3) (Santa Cruz Biotechnology), C15100066 (Diagenode) and 06–935 (Millipore). For this purpose, we used the ERα positive cell line MCF7 and performed ChIP-qPCR in biological duplicates **(S1 Fig)** to assess ERα binding at known target regions **(S1 Table)**.

The ChIP-qPCR comparison suggested that 06–935 (Millipore) and ab3575 (Abcam) could successfully enrich ERα-bound chromatin at these selected loci and could therefore substitute for sc-543. We performed ChIP-seq to compare these three antibodies in MCF7 cells using IgG as a negative control. ERα ChIP-seq was performed in at least duplicates for each condition, using the same batch of chromatin, to ensure that antibodies could be directly compared. In addition, we included the ERα negative MDA-MB-231 cell line in order to assess non-specific binding by these antibodies. For MDA-MB-231, ChIP-seq was performed in biological triplicates.

**Fig 1:**
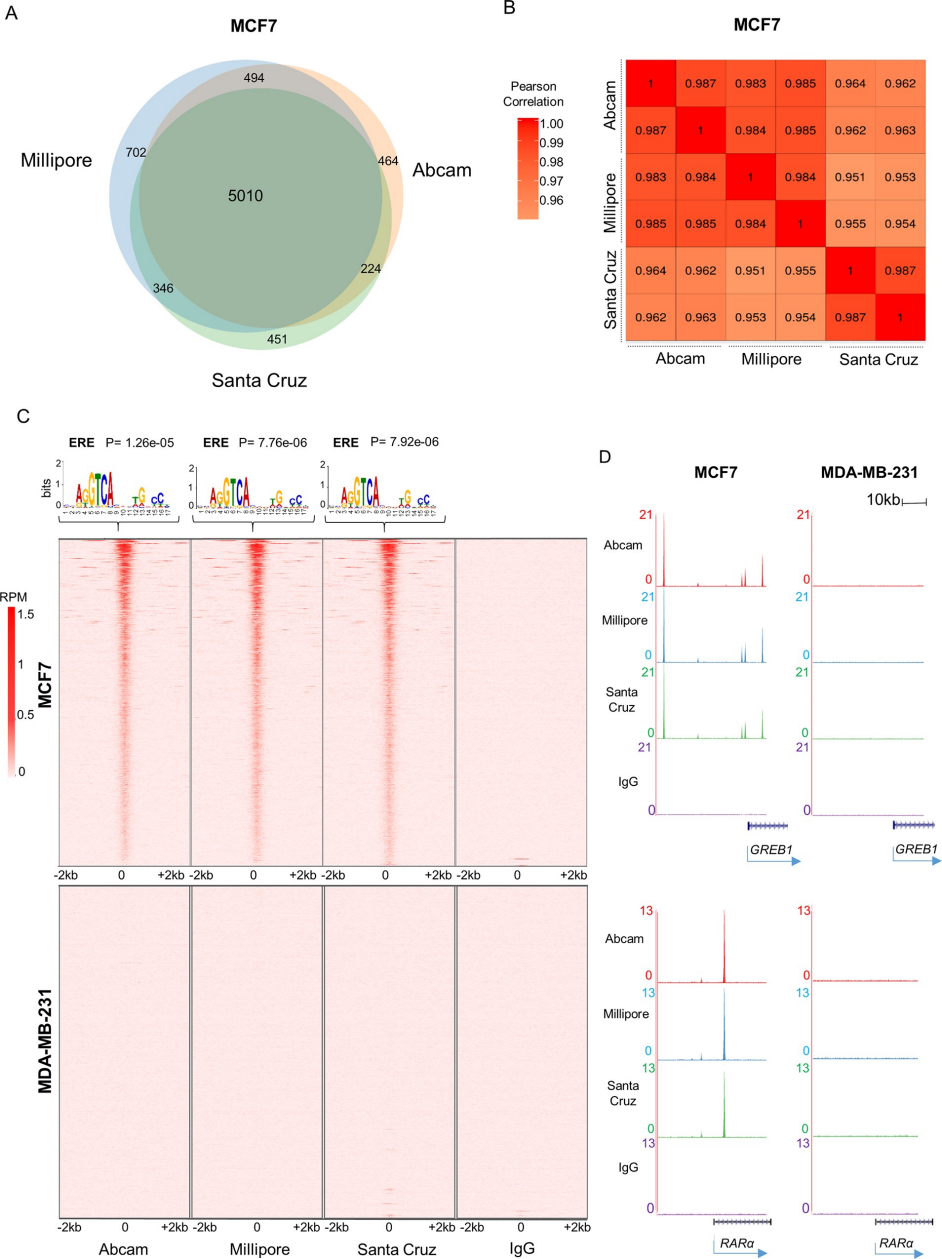
ChIP-seq comparison between Santa Cruz (sc-543), Millipore (06–935) and Abcam (ab3575) antibodies. A) Venn diagram showing the overlap between ERα binding sites for Santa Cruz (sc-543), Millipore (06–935) and Abcam (ab3575) antibodies in MCF7 cells. B) Pearson's correlation between each replicate of all three antibodies in MCF7 cells. C) Top: De novo motif analysis of ERα binding sites using MEME. Bottom: Heatmap of total number of ERα binding sites identified in both technical replicates of MCF7, and in all three biological replicates for MDA-MB-231, respectively. D) Examples of ERα- bound regions. Tag densities are shown as reads per million.

We observed 6,031 ERα binding sites for sc-543 (Santa Cruz) antibody, 6,192 peaks for ab3575 (Abcam) and 6,552 for 06–935 (Millipore). Importantly, none of these binding sites were observed in the IgG negative control. The vast majority of sites identified in MCF7 cells by sc-543 overlapped with those detected by ab3575 and 06–935 **(Fig 1A)**. Consistently, we found a strong correlation between the binding intensities for the three antibodies, which was similar to the correlation between replicates for the same antibody **(Fig 1B)**. All three antibodies showed robust enrichment at binding sites compared to background and motif analysis identified the ERα response element (ERE) as highly significantly enriched at these sites (**Fig 1C**). Importantly, neither of the ab3575 and 06–935 antibodies showed any significant enrichment in the ERα negative cell line MDA-MB-231 **(Fig 1C)**. In total, one peak was detected in ER-negative cells using ab3575, two peaks for 06–935 and 124 binding sites for sc-543, confirming the specificity of the antibodies. Examples of ERα binding to previously described ERα binding sites[16, 31] are illustrated in **Fig 1D**. Taken together, this indicates that the ab3575 (Abcam) and 06–935 (Millipore) antibodies perform similarly to the sc-543 (Santa Cruz) antibody in ChIP-seq experiments, both in terms of sensitivity and specificity.

### Validation of 06–935 and ab3575 antibodies using RIME

We next sought to evaluate the performance of ab3575 (Abcam) and 06–935 (Millipore) in RIME experiments to directly compare with the sc-543 (Santa Cruz) antibody, which has previously been successfully used in RIME experiments to explore the ERα interactome[9, 16, 22]. To this end, we tested the 06–935, ab3575 and sc-543 antibodies in two technical replicates each using MCF7 cells. IgG controls were also analysed to discriminate specific associations from non-specific interaction events.

**Fig 2:**
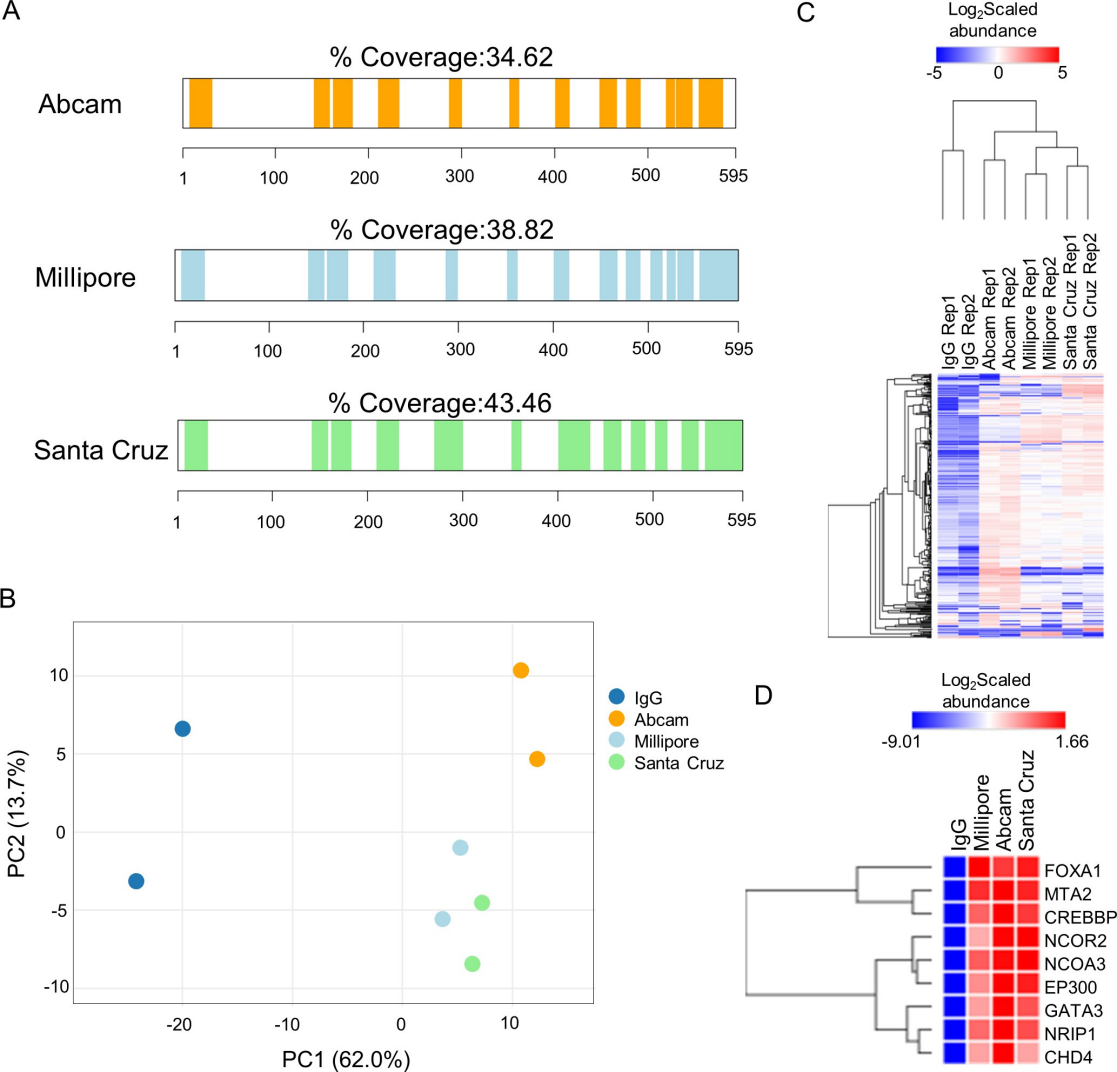
Comparison of RIME data between Santa Cruz (sc-543), Millipore (06–935) and Abcam (ab3575) antibodies. A) Protein sequence coverage of ERα achieved by the use of Abcam (ab3575), Millipore (06–935) and Santa Cruz (sc-543) antibodies in RIME. B) PCA plot of known ERα interactors (n = 319, BIOGRID and STRING databases) for the four different RIME pull-downs. C) Hierarchical clustering of the scaled intensities of known ERα interactors from BIOGRID and STRING databases (n = 319). D) Hierarchical clustering of well-characterized ERα interactors.

To evaluate the pull-down efficiencies, we compared the sequence coverage of the bait protein obtained by the different antibodies. ERα was identified with a similar number of peptides **(Fig 2A)** across the three different pull-downs, confirming that all three antibodies achieve efficient immunoprecipitation of the bait protein. Next, to compare the efficiency of the different antibodies to detect known ERα interactors, we used a label-free quantification method based on the Minora algorithm implemented in Proteome Discoverer 2.2 software **(S2 File)**. The PCA plot using intensities of known ERα-associated proteins (n = 319, BIOGRID and STRING databases) across all four samples revealed a good separation between the ERα RIME samples and the IgG controls, indicative of high specificity of all antibodies (**Fig 2B)**. Importantly, we identify only minor differences between the three antibodies, suggesting that they all efficiently pull down known ERα-associated proteins (**Fig 2B and 2C)**. Specifically, amongst the known ERα interactors we identified FOXA1, GATA3 and members of the p160 family that were all highly enriched by all three antibodies (**Fig 2D)**. Taken together, the three ERα antibodies perform similarly in RIME experiments, enriching for well-known key ERα interactors.

## Conclusions

Genome-wide analyses of ERα-chromatin binding sites using ChIP-based methods have exponentially increased our knowledge of the role of ERα in breast cancer. Most of the published ChIP-seq and RIME studies for ERα have been performed using the sc-543 antibody from Santa Cruz Biotechnology[13, 16, 17, 19–21, 32] and the quality and specificity of sc-543 has made it the ‘golden standard’ for immunoprecipitation experiments. However, this antibody has recently been discontinued, which has significantly impacted our ability to study ERα biology. Here, we have assessed commercially available alternative antibodies. We demonstrate using ChIP-seq and RIME that the two antibodies 06–935 (Millipore) and ab3575 (Abcam) perform similarly to sc-543, in terms of sensitivity and specificity. We therefore propose that these antibodies can replace the sc-543 antibody for immunoprecipitation-based experiments such as ChIP-seq and RIME to explore ERα function.

## Supporting information

S1 Fig ERα antibody comparison by ChIP-qPCR. ChIP-qPCR analysis for ERα known binding sites was performed in MCF7 cells in biological duplicates. Results are shown as arbitrary units. Antibodies used: sc-543 (Santa Cruz Biotechnology), ab80922 (Abcam), ab3575 (Abcam), sc-514857 (C-3) (Santa Cruz Biotechnology), C15100066 (Diagenode) and 6–935 (EMD Millipore).
(TIF)

S1 File Main steps of the ChIP-seq analysis. The file provides details for the main steps of the Bioinformatic analysis of the ChIP-seq data.
(PDF)

S2 File Quantitative proteomics analysis results. The file contains the protein intensities across all the different RIME samples based on a label free quantification method using the Minora algorithm in Proteome Discoverer 2.2.
(XLSX)

S1 Table ChIP-qPCR primers. Table listing the primers used for the ChIP-qPCR experiment.
(DOCX)
