# Supplementary figures and images for: Identification of ChIP-seq and RIME grade antibodies for Estrogen Receptor alpha
Source: PLoS One. 2019 Apr 10;14(4):e0215340. doi: 10.1371/journal.pone.0215340 (PMC6457525; doi:10.1371/journal.pone.0215340)

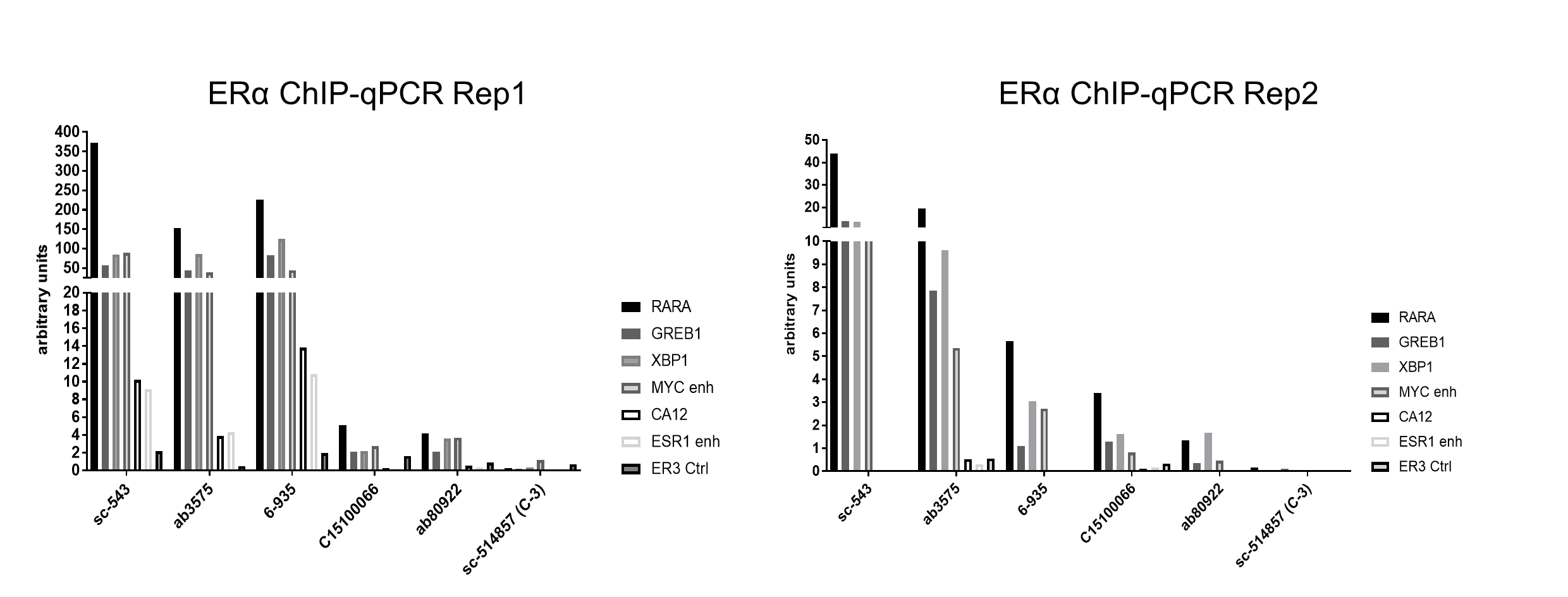

Supplement: S1 Fig — ChIP-qPCR analysis for ERα known binding sites was performed in MCF7 cells in biological duplicates. Results are shown as arbitrary units. Antibodies used: sc-543 (Santa Cruz Biotechnology), ab80922 (Abcam), ab3575 (Abcam), sc-514857 (C-3) (Santa Cruz Biotechnology), C15100066 (Diagenode) and 6–935 (EMD Millipore). (TIF) [file pone.0215340.s001.tif]
