# Supplementary material for: Identification of ChIP-seq and RIME grade antibodies for Estrogen Receptor alpha
Source: PLoS One. 2019 Apr 10;14(4):e0215340. doi: 10.1371/journal.pone.0215340 (PMC6457525; doi:10.1371/journal.pone.0215340)
Supplement: S1 File — The file provides details for the main steps of the Bioinformatic analysis of the ChIP-seq data. (PDF) [file pone.0215340.s002.pdf]

Bioinformatics Supplementary Methods

Introduction

This document provides details for the main steps of the bioinformatic analysis of the data. Minor steps, such as indexing bam files with `samtools`, have been omitted, but the detail contained herein should be sufficient to replicate the analysis documented in the main paper starting from the raw the fastq files.

The R scripts refered to in the code blocks below were included in the zip file with this file. R version 3.4.0 was used throughout. R package versions are listed in the relevant sections.

Generic input and output file names have been provided in each command line, e.g. `<InputFastq>`, in place of the specific sample filenames.

1. Alignment

Sequencing was carried out across 3 lanes on an Illumina HiSeq 4000. Fastq files from each lane were aligned separately. Alignment was carried out using `bwa` version 0.7.12[1] against the hg38 human reference genome downloaded from (UCSC) [http://hgdownload.cse.ucsc.edu/goldenPath/hg38/bigZips/hg38.fa.gz].

```
bwa aln <Input_Fastq> hg38.fa > <Aligned_sai>

bwa samse hg38.fa <Aligned_sai> <Input_Fastq> \
    | samtools view -h -b -o <Aligned_bam>
```

2. Merge lanes and mark duplicates

Lane-level aligned bam files were merged and duplicates marked using the `Picard` version 2.9.0 tool `MarkDuplicates`.

```
java --jar picard.jar MarkDuplicates \
    INPUT=<Aligned_bam_lane_1> \
    INPUT=<Aligned_bam_lane_2> \
    INPUT=<Aligned_bam_lane_3> \
    OUTPUT=<Sample_Bam> \
    METRICS_FILE=<Metrics_Output> \
    CREATE_INDEX="true"   \
    VALIDATION_STRINGENCY=SILENT
```

3. Generate greylists

The Bioconductor[2] package *GreyListChIP* [3] version 1.8.0 was used to identify regions of anomalous signal in the inputs for filtering.

The `hg38.sizes` file is simple two column karyotype file describing the size of each chromosome as described in the *GreyListChIP* documentation.

```
Rscript" --vanilla GenerateGreyList.R \
    --bamFile <Input_Bam_File> \
    --karyoFile hg38.sizes \
    --outputFile <Greylist_Bed_File> \
    --nCores 6
```

optparse - version 1.3.2
magrittr - version 1.5

Each input was used to generate a greylist. The final greylist used in filtering was the union of all four individual greylists. `bedtools` version 2.26.0 was used to merge the greylists.

```
cat *.greylist.bed \
| sort -V \
| bedtools merge \
    -i - \
    -d 2048 \
    > AllInputs.greylist.bed"
```

4. Filter bams

The sample level aligned bams were filtered using custom java classes according to the following rules:

Order	Filter	Action
1	Unmapped reads	Exclude reads with sam flag 0x004
2	Canoncial Chromosome	Exclude reads not aligned to chrosomes 1-22, X, Y
3	Blacklist	Exclude reads aligning to regions in blacklist
4	Greylist	Exclude reads aligning to regions in Greylist bed file
5	Mapping Quality	Exclude reads with a mapping quality less than 15

The blacklist file for hg38 was downloaded from http://mitra.stanford.edu/kundaje/akundaje/release/blacklists/hg38-human/hg38.blacklist.bed.gz

4. Merge unfilterd replicate bams

For each antibody and cell type combination the 3 replicate unfiltered bams were merged using the `Picard` version 2.9.0 tool `MergeSamFiles`.

```
java --jar picard.jar MergeSamFiles \
    INPUT=<Unfiltered_bam_rep_1> \
    INPUT=<Unfiltered_bam_rep_2> \
    INPUT=<Unfiltered_bam_rep_3> \
    OUTPUT=<Merged_Bam> \
    VALIDATION_STRINGENCY=SILENT
```

5. Merge filter replicate bams

For each antibody/input and cell type combination the 3 replicate filtered bams were merged after having reads marked as duplicates removed.

```
# remove duplicates
java --jar picard.jar MarkDuplicates \
      I=<Filtered_bam> \
      O=<Deduplicated_bam> \
      M=<Duplication_metrics_file> \
      REMOVE_DUPLICATES=true

# merge bams
```

```
java --jar picard.jar MergeSamFiles \
    INPUT=<Deduplicated_bam_rep_1> \
    INPUT=<Deduplicated_bam_rep_2> \
    INPUT=<Deduplicated_bam_rep_3> \
    OUTPUT=<Filtered_Bam> \
    VALIDATION_STRINGENCY=SILENT
```

Peak calling

Peak calling was carried out using `MACS2` version 2.1.1[4] using the merged filtered bam files.

```
"/home/sawle01/pipelines/myChIPSeqPipeline/pipelinesoftware/chipseq/el7/python-2.7/bin/macs2" callpeak \
    --treatment <SampleGroup_Filtered_Bam> \
    --control <Input_Bam_Filtered_Bam> \
    --gsize "2685753917" \
    --outdir <Output_Dir> \
    --name <SampleName> \
    --verbose 2 \
    --fix-bimodal \
    --extsize 200 \
    --qvalue 0.05 \
    --keep-dup all
```

Generate Venn Diagrams

The Bioconductor package *DiffBind* [5] was used to generate a consensus peak set between all MCF7 peak sets and to count the total reads associated with each peak in the unfiltered merged bam files. The R package *venn* has been used in this script to generate the venn diagram.

```
Rscript --vanilla MCF7_Venn_Data.R
```

Generate Heatmaps

The Bioconductor packages *genomation* and *ComplexHeatmap* were used to generate tag peak occupancy heatmaps. Tag counts were generated for all observed peaks using *genomation*.

```
Rscript --vanilla PlotHeatmaps.R
```

Motif Analysis

Motif analysis was carried out using the MEME Suite[6], specifically **AME**[7] and **MEME-ChIP**[8].

For each sample, sequences used for motif analysis were obtained by selecting (up to) the top 1000 peaks based on the q-value provided by MACS2 and then extracting the genomic sequence 500 bases up-stream and down-stream of the peak summit (1000 bases in total).

The Homo sapiens Comprehensive Model Collection (HOCOMOCO) [9] version 10, as provided on the MEME Suite website, was used as the reference.

Run Ame

```
# make alphabet file from motif database
meme2alph HOCOMOCOv10_HUMAN_mono_meme_format.meme alphabet.txt

# create shuffled control
fasta-shuffle-letters \
    -alph alphabet.txt \
    -kmer 2 \
    -tag -dinuc \
    -seed 1 \
    <InputSequenceFile> <ShuffledControl>

# run ame
ame -oc <ResultsDirectory> \
    --control <ShuffledControl>\
    <InputSequenceFile>\
    HOCOMOCOv10_HUMAN_mono_meme_format.meme
```

Run MEME-ChIP

```
meme-chip -db HOCOMOCOv10_HUMAN_mono_meme_format.meme \
    -oc <ResultsDirectory> \
    -meme-p 6 \
    -spamo-skip \
    -fimo-skip \
    HOCOMOCOv10_HUMAN_mono_meme_format.meme
```

References

1. Li H. and Durbin R. (2009) Fast and accurate short read alignment with Burrows-Wheeler Transform. Bioinformatics, 25:1754-60
2. Huber W. et al (2015) Orchestrating high-throughput genomic analysis with Bioconductor. Nature Methods, 12:115
3. Brown G (2018). GreyListChIP: Grey Lists – Mask Artefact Regions Based on ChIP Inputs. R package version 1.12.0.
4. Zhang et al. (2008) Model-based Analysis of ChIP-Seq (MACS). Genome Biol, 9:R137
5. Ross-Innes, C. S. et al . (2012). Differential oestrogen receptor binding is associated with clinical outcome in breast cancer. Nature 481:389-393.
6. Bailey T.L et al (2009) MEME SUITE: tools for motif discovery and searching. Nucleic Acids Research, 37:W202-W208
7. McLeay R.C. and Bailey T.L. (2010) Motif Enrichment Analysis: a unified framework and an evaluation on ChIP data. BMC Bioinformatics, 11:165
8. Machanick P. and Bailey T.L. (2011) MEME-ChIP: motif analysis of large DNA datasets. Bioinformatics 27(12):1696-1697
9. Kulakovskiy I.V. et al (2016) HOCOMOCO: expansion and enhancement of the collection of transcription factor binding sites models. Nucl. Acids Res. 44(D1):D116-D125
