# Supplementary material for: Identification of ChIP-seq and RIME grade antibodies for Estrogen Receptor alpha
Source: PLoS One. 2019 Apr 10;14(4):e0215340. doi: 10.1371/journal.pone.0215340 (PMC6457525; doi:10.1371/journal.pone.0215340)
Supplement: S1 Table — Table listing the primers used for the ChIP-qPCR experiment. (DOCX) [file pone.0215340.s004.docx]

| **Name** | **FWD primer** | **REV primer** |
| --- | --- | --- |
| XBP1 enh 1 | ATACTTGGCAGCCTGTGACC | GGTCCACAAAGCAGGAAAAA |
| GREB1 enh 3 | GAAGGGCAGAGCTGATAACG | GACCCAGTTGCCACACTTTT |
| RARA intron | GCTGGGTCCTCTGGCTGTTC | CCGGGATAAAGCCACTCCAA |
| MYC enh | GCTCTGGGCACACACATTGG | GGCTCACCCTTGCTGATGCT |
| ESR1 Enh 3 | GAAACAGCCCCAAATCTCAA | TTGTAGCCAGCAAGCAAATG |
| CA12 | GGAGGCGTAACCCCTGTGTG | ACGGCAAGGGACTTGCTGAC |
| ER3 Control | GCCACCAGCCTGCTTTCTGT | CGTGGATGGGTCCGAGAAAC |

**S1 Table**
